# Effect of colporrhaphy on the Sexual Dysfunction of women with pelvic organ prolapsed

**DOI:** 10.12669/pjms.291.2610

**Published:** 2013

**Authors:** Sholeh Shahghaibi, Sana Faizi, Fardin Gharibi

**Affiliations:** 1 Sholeh Shahghaibi, Asso. Prof. of Gynecology, Dept. of Gynecology, Faculty of Medicine, Kurdistan University of Medical Sciences, Sanandaj, Iran.; 2 Sana Faizi, General Practitioner, Kurdistan University of Medical Sciences, Sanandaj, Iran.; 3 Fardin Gharibi, MsPH Health Management, Vice Chancellor for Research Affairs, Kurdistan University of Medical Sciences, Sanandaj, Iran.

**Keywords:** Colporrhaphy, Pelvic organ prolapse, Sexual dysfunctions

## Abstract

***Objective:*** To evaluate the effect of colporrhaphy on sexual problems of women with pelvic organ prolapseis.

**Methodology:** Sixty patients with pelvic organ prolapse (cystocele - rectocele) in a quasi-experimental study before and after treatment were included. Presence of pelvic organ prolapseis was assessed by examination in the lithotomy position. Degree of prolapse was determined according to Pelvic organ Prolapse Quantification (POPQ). Their degree of prolapse was marked from 1 to 3 then was colporrhaphy performed. Follow up of patients for sexual satisfaction was performed three and six months after surgery by telephone and interviews. Data were analyzed by SPSS version 15, the McNamara test, T-test, absolute and relative frequency, mean and standard deviation.

***Results:*** In sixty (60) women who were studied the mean and standard deviation of pregnancy and delivery rate were 4.4 ±2.2 and 4±2.1 respectively. About 65% of patients were over 35 years of age, 88.3% of women had the history of vaginal delivery and 10% of them had both the history of vaginal delivery and cesarean section. Score of sexual desire abstaining from sex and orgasm due to vaginal bulge before, 3 months and 6 months after colporrhaphy was significant (p=0.0001), but of sexual arousal scores there was no differences between each other. Mean of sexual satisfaction before operation was 30.75±5.70 and three months after operation it was 33.77±4.24 and six months after operation 36.03±3.5 which according to T-Test there was significant (p=0.0001).

***Conclusion:*** Sexual desire, orgasm and sexual satisfaction increased after colporrhaphy, frequency of intercourse and sexual arousal remains unchanged. It appears that colporrhaphy reduce symptoms associated with prolapse which is leading to sexual dissatisfaction and improved sexual satisfaction.

## Introduction

 Prolapse, falling down of pelvic floor muscles is one of the gynecological problems. Pelvic visceral prolapse is described in three levels: 1-upper –anterior vaginal wall Prolapse, called cystocele 2-Posterior vaginal wall Prolapse, called rectocele, entrocele and 3-cervical prolapsed. Prolapse is the cause of dysfunction of pelvic organs, painful intercourse, sexual dysfunction and lack of sexual satisfaction of couples.^1^

 Incidence of prolapse is changed with parity, age and race. According to existing studies, about 50% of women suffered from some degree of prolapse after vaginal delivery, but only 10-20% of them have overt symptoms that its prevalence increases with age. Therefore 46% of over 60 years old women, suffer from symptomatic pelvic prolapse. High tension and tear during delivery and multiple deliveries seem to be the main predisposing obstetrics factors for symptomatic prolapse.^2^ Nygaard in his study in 2004 showed that 97.7% of postmenopausal women suffer from prolapsed.^3^ With increase of life expectancy and improving quality of life, it will be doubled in the next twenty-five years and has become a significant issue.^4^

 Sexual satisfaction is affected by many factors including; educational level and culture, job stress, struggle in couples relation, moral and sexual adaptation and also physical and mental disorders of couples. Pelvic floor muscle lameness is one of the physical problems. Lameness of the pelvic floor muscles, causes falling down pelvic organs (prolapsed), pelvic organ dysfunction, painful intercourse, back pain, sexual dysfunction and lack of sexual satisfaction.^1^^,^^5^ About 35% of patients with pelvic prolapse are suffering from sexual dysfunction and lack of sexual satisfaction.^6^

 A variety of approaches are available to correct pelvic organ prolapse, including; surgical and nonsurgical methods. Pessary (a device that is placed in the vagina to hold the uterus) Kegel exercises, physical therapy and hormone therapy are among non-surgical methods. Anterior and posterior colporrhaphy are among surgical methods.^1^ Due to the high success of the surgical treatment of pelvic prolapse symptoms, 85% of people who want definite treatment choose surgery to cure it. The frequency of colporrhaphy surgery in USA has been reported as four hundred thousand operations in year.^7^ Anterior colporrhaphy should only be done through the vagina. Surgical procedures that are performed through the abdomen are retropiocs including; Burch and Marshall Marchetti. Another method which is done both vaginal and abdominal is Needle suspension. Sling and artificial sphincter are other surgical methods.^8^ Benefits of anterior colporrhaphy is better patient compliance and anterior abdominal incision is not required, but its disadvantage is the lack of long-term success and upto 65% of the cases do not suffer from complications again.^9^

 In some studies as Barber and colleagues^10^ colporrhaphy improved sexual performance and increased sexual satisfaction, but in Rogers and colleagues’ study decrease in sexual satisfaction in the survey sample has been reported 3-6 months after surgery.^11^ Karimian and colleagues in their study have shown that women’s sexual problems resolved after colporrhaphy, but it had no effect on their sexual satisfaction.^5^

 The aim of this study was to evaluate the effect of colporrhaphy on pelvic organ prolapseis treatment and its effect to improve women’s sexual satisfaction.

## Methodology

 This study was conducted on sixty women with pelvic organ prolapseis who were referred to the clinic of Besat hospital in 2008-2009. The patients were married, their age ranges were 20-49 years and they had husband and children. Presence of pelvic organ prolapseis was assessed by examination in the lithotomy position. Degree of prolapse was determined according to Pelvic organ Prolapse Quantification (POPQ). Their degree of prolapse was marked from 1 to 3. Patients who were divorce had pelvic and breast surgeries, drug abuse, chronic diseases (diabetes, blood pressure and heart disease), and husband’s sexual problems, urinary and genital infections were excluded from the study. This study was approved by the Medical Ethics Committee of the university.

 After obtaining informed consent from women who qualified to enter the study, a “Prolapse/Urinary Incontinence Sexual Questionnaire “(PISQ) was completed for them. Then Colporrhaphy was performed by three gynecologists who employed the same approach in anterior, posterior and anterior-posterior colporrhaphy. Patients were evaluated for surgical complications (hematoma, bleeding, urinary retention) two days after surgery, and none of the mentioned complications were observed in any patient. Follow up of patients for prolapse related symptoms and sexual satisfaction were performed three and six months after surgery by telephone and interviews. Data were analyzed by SPSS version 15, the McNamara test, T-test, absolute and relative frequency, mean and standard deviation.

## Results

 In sixty (60) women who were studied the mean and standard deviation of pregnancy and delivery rate were 4 / 4 ± 2/2 and 4± 2/1 respectively. About 65% of patients were over 35 years of age. As regards education 51.7% were illiterate and only 5% had high school and college education. Of body mass in 38.3% overweight and in 33.3% obesity was seen. Body physical activity of women who were studied showed that 40% was high and 58.3% was medium. 88.3% of women had the history of vaginal delivery and 10% of them had both vaginal delivery and cesarean section (Table-I).

**Table-I:** Demographic characteristics of women

*Features*	*Mood*	*No.*	*Percent*
Age group	35 years and less	21	35
	More than 35	39	65
Education	Illiterate	31	51.7
	Elementary	15	25
	Secondary	11	18.3
	High school and over	3	5
BMI	Normal	17	28.3
	Overweight	23	38.4
	obesity	20	33.3
Activity Rate	high	24	40
	medium	35	58.3
	Low	1	1.7
Labor Method	vaginal	53	88.3
	Cesarean Section	1	4.7
	Both	6	10

 Sexual satisfaction questionnaire results showed that sexual desire score, abstaining from sex and orgasm due to vaginal bulge before 3 months and 6 months after Colporrhaphy was significant (p<0.05). However, there was no difference as regards scores of sexual arousal (Table-II).

**Table-II:** Comparison of mean of sexual activity scale before with 3 and 6 months after colporrhaphy in women with pelvic organ prolapseis

*Statement*	*Follow up time*	*Mean*	*SD*	P*
Sexual Desire	Before surgery	1.72	0.92	0.002
	After 3 months	2.10	0.73	
	After 6 months	2.22	0.71	
Abstaining from sex because of vaginal bulge	Before surgery	2.50	1.34	0.0001
	After 3 months	3.37	0.58	
	After 6 months	3.60	0.58	
Sexual arousal	Before surgery	3.15	0.97	0.433
	After 3 months	3.27	0.73	
	After 6 months	3.33	0.60	
Orgasm	Before surgery	2.08	0.88	0.0001
	After 3 months	2.27	0.71	
	After 6 months	3.33	0.60	

One-Way ANOVA

 Sexual desire score before Colporrhaphy was 1.72 three month after Colporrhaphy was 2.1 and six month after Colporrhaphy was 2.22 which is significant (p=.002). Abstaining from sex score because of vaginal bulge before Colporrhaphy, three months and six months after surgery respectively was, 5.2, 37.3 and 6.3 (p=.0001). Regarding orgasm score in women who were studied it was statistically significant before, three and six months after Colporrhaphy (p=.0001). Sexual arousal score before Colporrhaphy was 3.15 three months after was 3.27 and six months after was 3.33 which had no significance (Table-II).

 Comparison of preoperative dispareunia with 3 months after Colporrhaphy showed that 42.9% of women suffering from dispareunia were doing well and after six months it improved to 53.6%. In 28.1% of women who had no dispareunia before Colporrhaphy had this complication and six months after it reduced to 25% which according to McNamara test was not significant (Table-III).

**Table-III:** Comparison of dysparrnuia before with 3 and 6 months after colporrhaphy in women with pelvic organ prolapse

*After surgery*	*After 3 months*		*After 6 months*	
Before surgery	Yes (%)	No (%)	Yes (%)	No (%)
Yes (%)	16(57.1)	12(42.9)	13(46.4)	15(53.6)
No (%)	9(28.1)	23(78.1)	8(25.0)	24(75.0)
P*	0.36		0.21	

Mc NemarTest

 Mean of sexual satisfaction before operation was 30.75±5.70 three months after operation was 33.77±4.24 and six months after operation was 36.03±3.5 which according to T-Test was significant (p=.0001) (Table-IV).

**Table-IV:** Comparison of mean of sexual satisfaction scale before with 3 and 6 months after colporrhaphy in women with pelvic organ prolapseis

*Group*	*df*	*Mean & SD*	*Mean Difference & confidence interval*	*t*	*P*
Before surgery	118	30.78±5.70	3.02(2.3:29.74)	8.34	0.0001
After 3 months		33.77 ±4.24			
Before surgery	118	30.78±5.70	5.28(4.6:31.21)	11.08	0.0001
After 6 months		36.03± 3.5			

## Discussion

 This study showed that sexual satisfaction of women during the three and six months follow-up than before surgery had increased (p<0.05). Average score of sexual desire and orgasm during the six month follow up has an ascending trend, but rate of sexual arousal has not changed. Fear of herniation of vagina during intercourse, as a factor in patient refusal of sexual relations and Sexual dissatisfaction during the three and six month follow-up was also reduced.

 Due to decrease in the number of patients who suffered from dysparenuia before colporrhaphy and initiation in patients who had not dysparenuia before surgery dysparenuia improvement was not seen (P=0.21). During six months follow up in 25% of patients who before surgery had no dysparenuia this complication was observed.

 In Porter and colleague’s study^12^ during the six months follow up in 73% of patients dysparenuia eliminated 19% became worse and in 11% initiated. In Weber and colleagues study^13^ dysparenuia has been reported in 8% of women before and 19% of them six months after surgery. In Gungor and colleagues study in Turkey^14^ in 18% of patients during a six-month follow-up dysparenuia became worse and caused sexual dissatisfaction. In Nieminen study^15^ patients dysparenuia compared to before surgery had no significant difference, but in the group treated by anterior colporrhaphy and polypropylene mesh it was reduced significantly. With these results it appears that colporrhaphy has a role in creating dysparenuia after surgery which are consistent with the findings of our study.

 In Kariman’ study^5^ the number of those who experienced orgasm, frequency of intercourse and sexual desire had increased during three months follow up after surgery but sexual satisfaction had no significant differences with before surgery. Perhaps differences in sexual satisfaction between the two studies is due to longer follow-up in our study.

 In Gungor study^14^ Six months after surgery with anterior colporrhaphy and colpoprinorhaphy sexual satisfaction in 66% of patients improved and in 20% it became worse. Levels of sexual satisfaction after surgery increased and this is consistent with our study. In Nieminen study sexual desire, frequency of intercourse, ability to achieve orgasm was unchanged and sexual satisfaction during 24 months follow up was not statistically significant than before surgery (p=0.83).^15^

 In Nieminen study the mean age was 66 years whereas in our study patients were under 50 years of age and were younger than that study and it may be the reason for the difference in sexual desire, orgasm and sexual satisfaction results. In Pauls and colleagues study during six-month follow-up after surgery sexual function had no significant differences with before surgery.^16^

 In Porter study^12^ there was no considerable differences in sexual desire, ability to orgasm and intercourse frequency and sexual satisfaction before and six months after surgery. Patients age was different as compared to our study and they used a specific approach in surgery which was different with posterior Colporrhaphy in our study and it may be the reason of differences in two studies findings.

 In Komesu and colleagues study in USA^17^ sexual satisfaction of patients who had undergone posterior repair was increased and is consistent with our study. In Barber and colleagues study a smaller number of patients with pelvic organ prolapse mentioned the negative impact of vaginal prolapse during intercourse six months after surgery (9% as against 29% before surgery).^10^

 Porter and colleagues in their study had not seen any changes in sexual desire; orgasm and sexual satisfaction but patients’ dyspareunia had improved.^12^ But in Kahn study in London in 1 to 6 years follow up after colporrhaphy sexual dysfunction in women increased from 18% to 27%.^18^

 We concluded that during sexual desire, orgasm and sexual satisfaction increased after colporrhaphy, frequency of intercourse and sexual arousal remains unchanged. It appears that colporrhaphy reduce symptoms associated with prolapse which is leading to sexual dissatisfaction and improved sexual satisfaction.
